# Synthesis of *N*-Tetradecyl-1,10-phenathrolinium-Based New Salts for Biological Applications

**DOI:** 10.1155/2018/8097483

**Published:** 2018-07-17

**Authors:** Atakilt Abebe, Minaleshewa Atlabachew, Misganaw Liyew, Elsabet Ferede

**Affiliations:** ^1^Chemistry Department, Science College, Bahir Dar University, Bahir Dar, Ethiopia; ^2^Biology Department, Science College, Microbiology Research Laboratory, Bahir Dar University, Bahir Dar, Ethiopia

## Abstract

New organic salts were synthesized by quaternizing 1,10-phenanthroline using 1-bromotetradecane. The first step yielded an organic salt of formula [C_26_H_37_N_2_]Br. Anion exchange reaction using Li[(CF_3_SO_2_)_2_N] resulted in a more stable salt of formula [C_26_H_37_N_2_][(CF_3_SO_2_)_2_N]. The organic salts were investigated by spectrometry (^1^H, ^13^C, ^19^F NMR, X-ray photoelectron spectroscopy (XPS), UV-Vis, and matrix-assisted laser desorption/ionization mass spectroscopy (MALDI MS), CHNSBr elemental analysis, and thermal analysis (TGA and DSC). The thermal characterization showed the melting and decomposition points of [C_26_H_37_N_2_][(CF_3_SO_2_)_2_N] to be 48°C and 290°C, respectively, which indicates it is an ionic liquid with large liquidus range. The biological activities of the salts were investigated against two Gram-positive (*Staphylococcus aureus* and *Streptococcus pyogenes*) and two Gram-negative (*Escherichia coli* and *Klebsiella pneumoniae*) bacteria, and they are found to be active against all of them. They were compared with [Cu(1,10-phenanthroline)_2_Cl]Cl. They are found more active against the Gram-negative bacteria. The salts demonstrated minimum inhibitory concentration as low as 50 *µ*g/L. These results suggest the synthesized salts can be considered as a better alternative to certain transition metal complex drugs. This minimizes the concern of introducing metal ions into the organism.

## 1. Introduction

Nucleic acids play a central role in critical cellular processes including cell division and protein expression [1]. Therefore, they are very attractive targets for small molecule therapeutics [2]. Most biological processes are reliant upon molecular recognition and the reversible interactions of one set of molecules with another [3, 4]. Failure in these activities results in malfunctions in cell replication or gene expression. This could be controlled by modulating nucleic acid activity through use of sequence- or structure-specific drugs. Such compounds would have a direct and beneficial role in the treatment of major diseases [5]. Compounds that have the potential to be clinically useful are normally either intercalators, groove binders, or capable of external electrostatic interaction to DNA [6]. In this regard, aromatic molecules with rigid planar or approximately planar aromatic ring structure systems are the primary choices [7–9]. One such biologically active moiety is 1,10-phenanthroline. However, molecular 1,10-phenanthroline removes metal ions from biological systems using its conveniently placed coordinating two nitrogen atoms. This is expressed by the inhibition of metalloenzymes removing metal ions required for catalytic activity [10, 11]. Therefore, one present strategy in the field of pharmacological research is coordinating 1,10-phenanthroline with transition metal ions to make its nitrogen atoms unavailable for denaturation [12–15]. Furthermore, this activity enhances the medicinal activity of transition metal complexes [16, 17]. Based on this, many transition metal complexes of 1,10-phenanthroline have been found exhibiting numerous biological activities such as antiviral [18, 19], anti-inflammatory, antitumor [20], anti-Candida [21], antimycobacterial [22], and antimicrobial [23] activity upon binding to DNA. For example, [bis(1,10-phenanthroline)L_2_copper(II)] (**L** = *cis*-5-norbornene-endo-2, 3-dicarboxylic acid; 2,2′-bipyridine; dicyanamide, adenine, and thymine) is well known for its numerous biological activities such as anti-Candida [21], antimycobacterial, and antimicrobial [23] activity. Its activity is believed to emerge from an efficient DNA cleavage activity [24]. However, their rigid three-dimensional structures allow intercalation of the coordinated 1,10-phenanthroline only partially in the major groove of the DNA strand. The latter diminishes the activity of the complex [25, 26].

In this work, an alternative to the employment of transition metal complexes of 1,10-phenanthroline for transportation of 1,10-phenanthroline to the cellular target is described. This is achieved by quaternizing one of the nitrogen atoms in 1,10-phenanthroline using 1-bromo-tetradecane which produces *N*-tetradecyl-1,10-phenanthrolinium bromide organic salt. This results in an amphiphilic, serpent-like, flat head cation. The amphiphilicity is a consequence of the combined effects of the hydrophobic alkyl chain and the positive charge delocalized throughout the aromatic ring portion of 1,10-phenanthroline [27]. Moreover, its required flat structure is retained intact. This possibly results a complete intercalation in both the major and minor grooves of DNA. The bromide is exchanged with the lipophilic bis(trifluoromethylsulfonyl) imide, [(CF_3_SO_2_)_2_N]^−^, which compounds the penetration of the salt through the lipophilic cell membrane and cell wall which maximizes the biological activity of the salt. Furthermore, the biological activities of these organic salts will be compared with chloro-bis(1,10-phenanthroline)copper(II) chloride. In this comparison, the number of 1,10-phenanthroline moieties in both systems would be made equal. The synthesis of the organic salt from 1,10-phenanthroline is described in the experimental section. The synthesis of the copper complex was carried out following reported procedures [28].

## 2. Experimental

### 2.1. Materials and Methods

Chemicals used in this work include 1,10-phenanthroline monohydrate and lithium bis(trifluoromethanesulfonyl) imide obtained from Alfa Aesar. 1-Bromotetradecane, 1,4-dioxane, and all the solvents used were obtained from Sigma-Aldrich and used as received.

The prepared compounds were characterized by their ^1^H NMR, ^13^C NMR, and ^19^F NMR spectra which were recorded on a 400 MHz Bruker 400 Ultrashield NMR with operating frequencies 270 MHz (^1^H), 68 MHz (^13^C), and 376 MHz (^19^F). Chemical shifts (*δ*) are reported in parts per million (ppm) with reference to residual traces in the commercial deuterated solvent, dimethyl sulfoxide (CD_3_)_2_SO (*δ*_H_ 2.54, (*δ*_C_ 40.45)), at ambient temperature. Coupling constants (*J*) are given in Hz. The electronic environment of the component atoms of the cation, [C_26_H_37_N_2_]^1+^, and the anion, bistrifluorosulfonyl imide ([(CF_3_SO_2_)_2_N]^−^), were investigated using ultra-high vacuum (UHV) characterization using X-ray photoelectron spectroscopy (XPS). We followed the method of Men et al. [29]. The X-ray photoelectron spectra were recorded using a Kratos Axis Ultra spectrometer employing a focused, monochromated Al K*α* source (*hν* = 1486.6 eV), hybrid (magnetic/electrostatic) optics, hemispherical analyzer, and a multichannel plate and delay line detector (DLD) with an X-ray incident angle of 0° (relative to the surface normal). The spectrum was processed without charge correction. The information depth (ID) of these experiments may be defined as the depth, within the sample, from which 95% of the measured signal will originate. ID is assumed to vary mainly with cos *θ*, where *θ* is the electron emission angle relative to the surface normal. ID = 7–9 nm and the data obtained may be considered as a representative of the bulk composition. X-ray gun power was set to 100 W. All spectra were recorded using an entrance aperture of 300 × 700 *μ*m with pass energy of 80 eV for survey spectra and 20 eV for high-resolution spectra. The instrument sensitivity was 7.5 × 10^5^ counts·s^−1^ while measuring the Ag 3d_5/2_ photoemission peak for a clean Ag sample recorded at a pass energy of 20 eV and 450 W emission power. Ag 3d_5/2_ full width at half maximum (FWHM) was 0.55 eV for the same instrument settings. Binding energy calibration was made using Au 4f_7/2_ (83.96 eV), Ag 3d_5/2_ (368.21 eV), and Cu 2p_3/2_ (932.62 eV). The absolute error in the acquisition of binding energies is ±0.1 eV, as quoted by the instrument manufacturer (Kratos); consequently, any binding energy within 0.1 eV can be considered the same, within the experimental error. The absorption wave length in the UV-Vis was recorded using Cary 60, version 2.00, in the range 800 to 200 nm, with the UV-Vis scan rate 600 nm/min taking 0.01 mM solution. CHNS elemental analyses were performed with a Flash EA 1112 elemental analyzer (Thermo Quest) taking 15 mg sample. Bromide estimation was conducted taking 100 mg sample dissolved in 40 mL distilled water. Excess AgNO_3_ solution was added for the formation of silver bromide (AgBr) precipitate. Then the cruddy white precipitate formed was filtered, dried in an oven, and the amount of bromide was calculated from the weight difference. Matrix-assisted laser desorption/ionization mass spectrometry (MALDI-MS) in the reflectron mode was performed in a Bruker Ultraflex III for the investigation of the cation and anions. The matrix used was *trans*-2[3-(4-*tert*-butylphenyl)-2-methyl-2-propenylidene] malononitrile, commonly referred to as DCTB. The sample solution and the DCTB in acetonitrile were mixed together to give about 10 : 1 excess of matrix. 0.5 *µ*L of this mixture was spotted onto a stainless steel target plate and allowed to evaporate to dryness before introduction into the mass spectrometer. The laser operates at a wavelength of 337 nm. Calibration of the data was performed in the FlexControl software.

### 2.2. Synthesis of [C_26_H_37_N_2_]Br

The coordinated water in 1,10-phenanthroline monohydrate was removed heating in an oven at 105°C for 2 h. 1-Bromotetradecane dissolved in 1,4-dioxane was added from a dropping funnel to a molar equivalent 1,4-dioxane solution of 1,10-phenanthroline in a 100 ml two-necked round-bottomed flask fitted to the reflux condenser and guarded from moisture using CaCl_2_, being stirred in an oil bath at 50°C for 36 h. The completion of the reaction was followed up using thin-layer chromatography (TLC). Gray precipitate was obtained and washed thoroughly with 1,4-dioxane three times.

### 2.3. ^1^H and ^13^C of C_26_H_37_N_2_]Br

^1^H NMR (270 MHz, DMSO-*d*_6_) *δ* ppm: 0.81 (s, 3H), 1.19 (s, 18H), 1.34 (br. s., 2H), 1.48 (br. s., 2H), 2.07 (s, 2H), 5.89 (br. s., 2H), 8.07 (s, 1H), 8.43 (s, 3H), 8.80 (s, 1H), 9.27 (s, 1H), 9.44 (dd, *J* = 8.19, 1.31 Hz, 1H), and 9.70 (dd, *J* = 5.92, 1.38 Hz, 1H).

^13^C NMR (68 MHz, DMSO-*d*_6_) *δ* ppm: 14.49 (s, 1C), 22.64 (s, 1C), 26.27 (s, 1C), 29.11 (s, 1C), 29.25 (s, 1C), 29.44 (s, 1C), 29.58 (s, 2C), 31.57 (s, 1C), 31.84 (s, 1C), 63.92 (s, 1C), 125.19 (s, 1C), 125.91 (s, 1C), 127.72 (s, 1C), 131.10 (s, 1C), 132.23 (s, 1C), 133.16 (s, 1C), 136.94 (s, 1C), 138.52 (s, 1C), 140.15 (s, 1C), 147.59 (s, 1C), 150.45 (s, 1C), and 151.46 (s, 1C).

Spectra are indicated in Supporting information 1(a) and 1(b).

### 2.4. Synthesis of [C_26_H_37_N_2_][(CF_3_SO_2_)_2_N]

**Scheme 1:**
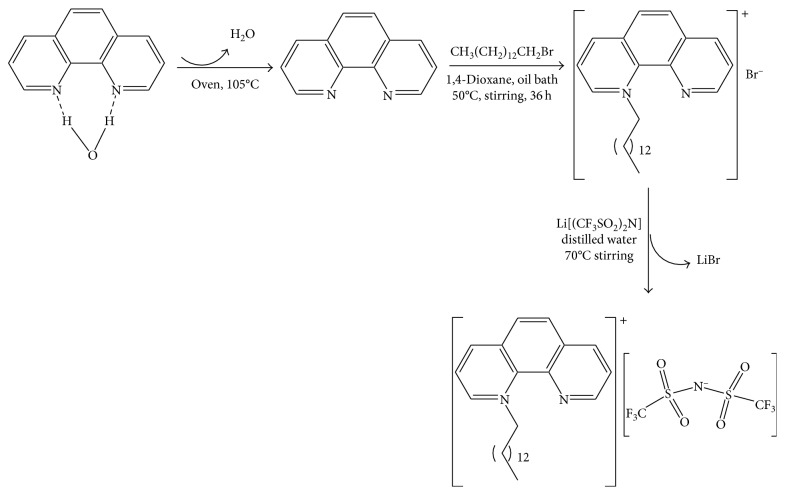
Synthesis path of the salts.

The halide anion was exchanged with (CF_3_SO_2_)_2_N^−^ by dissolving [C_26_H_37_N_2_]Br in deionized water in round-bottomed flask at 70°C to which slightly excess from equimolar amount solution of LiN(CF_3_SO_2_)_2_ was added dropwise. A separate phase viscous liquid following the formation of white suspension was formed. The viscous liquid was decanted, washed thoroughly at 70°C, and dried in vacuum. The viscous mass was solidified after several days. The path of the synthesis is indicated in Scheme 1.

### 2.5. ^1^H, ^13^C, and ^19^F NMR of [C_26_H_37_N_2_][(CF_3_SO_2_)_2_N]

^1^H NMR (270 MHz, DMSO-*d*_6_) *δ* ppm: 0.83 (t, *J* = 6.20 Hz, 3H), 1.21 (s, 18H), 1.35 (d, *J* = 6.47 Hz, 2H), 1.52 (br. s., 2H), 2.06 (d, *J* = 7.71 Hz, 2H), 5.80–5.98 (m, 2H), 7.99–8.11 (m, 1H), 8.32–8.54 (m, 3H), 8.74–8.86 (m, 1H), 9.30 (dd, *J* = 4.06, 1.58 Hz, 1H), 9.39 (d, *J* = 7.71 Hz, 1H), and 9.62 (d, *J* = 5.65 Hz, 1H).

^13^C NMR (68 MHz, DMSO-*d*_6_) *δ* ppm: 14.47 (s, 1C), 22.63 (s, 1C), 26.28 (s, 1C), 28.43–29.99 (m, 4C), 31.41–32.69 (m, 2C), 64.01 (s, 1C), 112.92–127.15 (q, 1C), 125.14 (s, 1C), 125.22–126.21 (m, 1C), 126.92–127.35 (m, 1C), 127.77–128.34 (m, 1C), 130.47–131.60 (m, 1C), 132.03–132.74 (m, 1C), 133.20 (s, 1C), 136.71–137.71 (m, 1C), 138.13–139.12 (m, 1C), 140.19 (s, 1C), 147.58 (s, 1C), 150.19–151.05 (m, 1C), and 151.39 (s, 1C).

Spectra are indicated in Supporting information 1(c) and 1(d).

^19^F NMR (376 MHz, DMSO-*d*_6_) *δ* ppm: −78.75 (s, 1 F).

Spectra are indicated in Supporting information 1(e).

### 2.6. Antibacterial Activity Testing

The organic salts were evaluated for *in vitro* antibacterial activities against strains of two Gram-positive (*S. aureus* and *S. pyogenes*) and two Gram-negative (*E. coli* and *K. pneumoniae*) bacteria. We followed the methods of Lawal et al. [30]. The bacterial strains were maintained in the appropriate blood agar base at 4°C. Antibiotic disc (gentamicin 10 *μ*g) was used as a reference. The minimum inhibitory concentration (MIC) against each bacterium was determined by preparing ethanolic solutions of different concentrations of the salts by serial dilution (50 *μ*g/mL, 75 *μ*g/mL, 100 *μ*g/mL, 125 *μ*g/mL, 150 *μ*g/mL, 175 *μ*g/mL, and 200 *μ*g/mL).

## 3. Results and Discussions

The salts are stable in air. They are soluble methanol, ethanol, acetonitrile, acetone, dichloromethane, and DMSO. The bromide salt dissolves in water as well. Elemental analyses value is in agreement with the assigned formulae. The elemental analysis values are given in Table 1.

### 3.1. ^1^H, ^13^C, and ^19^F NMR Results

1,10-Phenanthroline is a symmetric molecule that shows four types of protons and six types of carbon atoms in the aromatic region of its ^1^H NMR and ^13^C NMR spectra, respectively. However, following the quaternization reaction, it loses its symmetry evident from the appearance of eight types of protons and twelve types of carbons in this region. This new characteristic feature helps in the identification of the new salt. Moreover, the upfield appearance of fourteen types of protons and fourteen types of carbons and the number of alkyl protons of each type identified using their integration taking aromatic protons as references is a strong confirmation for the occurrence of quaternization (Supporting information 1(a)–1(d)). However, the possibility of di-quaternization of 1,10-phenanthroline is ruled out due to the steric hindrance of one of the nitrogen atoms after the first quaternization [31]. The successful anion exchange performed to get the intended salt is evident from the appearance of four new peaks in ^13^C NMR at *δ* ppm 112.92–127.15 assignable to the carbon in [(CF_3_SO_2_)_2_N]^−^ (Supporting information 1(d)). It is quartet because of the coupling with the bonded fluorine atoms. Moreover, the single strong peak signaled in the ^19^F NMR is an additional confirmation for the purity of the salt and the successful anion exchange reaction (Supporting information 1(e)).

### 3.2. XP Spectroscopy Results

**Figure 1:**
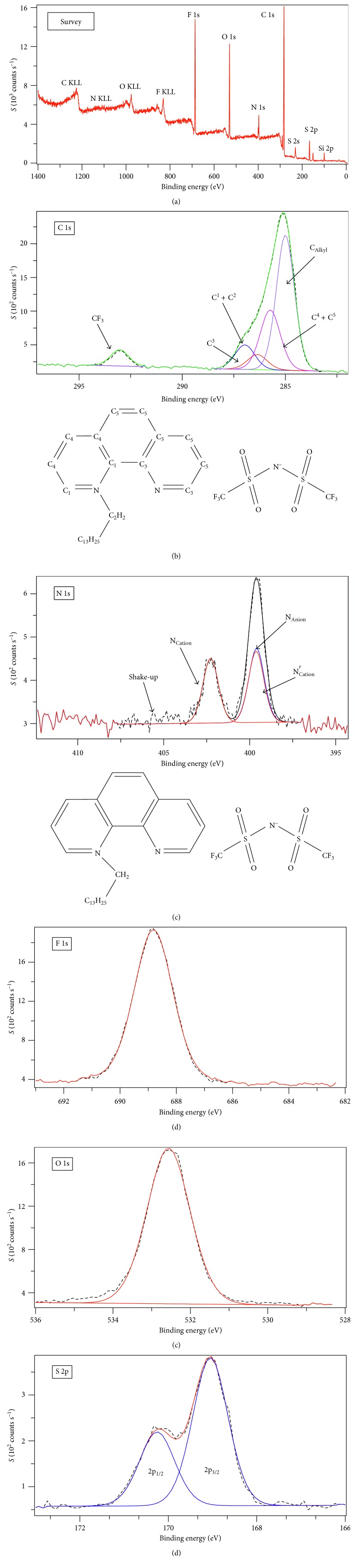
X-ray photoelectron spectrum recorded for (a) survey, (b) wide scan for C1s, (c) wide scan for N1s, (d) wide scan for F1s, (e) wide scan for O1s, and (f) wide scan for S2p of [C_26_H_37_N_2_][(CF_3_SO_2_)_2_N].

X-ray photoelectron spectroscopy is very reliable in confirming purity. The survey spectrum (Figure 1(a)) signaled only those elements expected from [C_26_H_37_N_2_][(CF_3_SO_2_)_2_N] in the appropriate percentage. This strongly supports the CHNS elemental analysis result.

### 3.3. Wide-Scan XPS Spectra: The Electronic Environments of the Component Atoms of [C_26_H_37_N_2_][(CF_3_SO_2_)_2_N]

In [C_26_H_37_N_2_][(CF_3_SO_2_)_2_N], there are 28 C atoms whose type of electronic environment is differentiated into five groups as CF_3_, C^1+2^, C^3^, C^4^ + C^5^, and C_Alkyl_. Their corresponding binding energies are 293.00, 286.90, 286.30, 285.70, and 285.00 eV, respectively (Figure 1(b)). The N 1s XP spectrum contains three characteristic peaks. The peak at higher binding energy, 402.20 eV, is assigned to the alkylated nitrogen of the cation, N_Cation_, while the peak at lower binding energy whose peak is nearly twice the former represents enveloped two nitrogenes, the unalkylated nitrogen, N′_Cation_ (399.52 eV), of the cation and that of the anion, **[(CF**_**3**_ **SO**_**2**_ **)**_**2**_ **N]** ^−^ (399.35 eV) (Figure 1(c)). Fluorine, oxygen, and sulfur of these compounds each show a single electronic environment (Figures 1(d)–1(f)). This is because of the obvious reason that the six fluorine atoms are chemically indistinguishable. This fact works the same for the four oxygen atoms and the two sulfur atoms. The sulfur signal is a doublet due to spin-orbit splitting.

### 3.4. Mass Spectra

**Figure 2:**
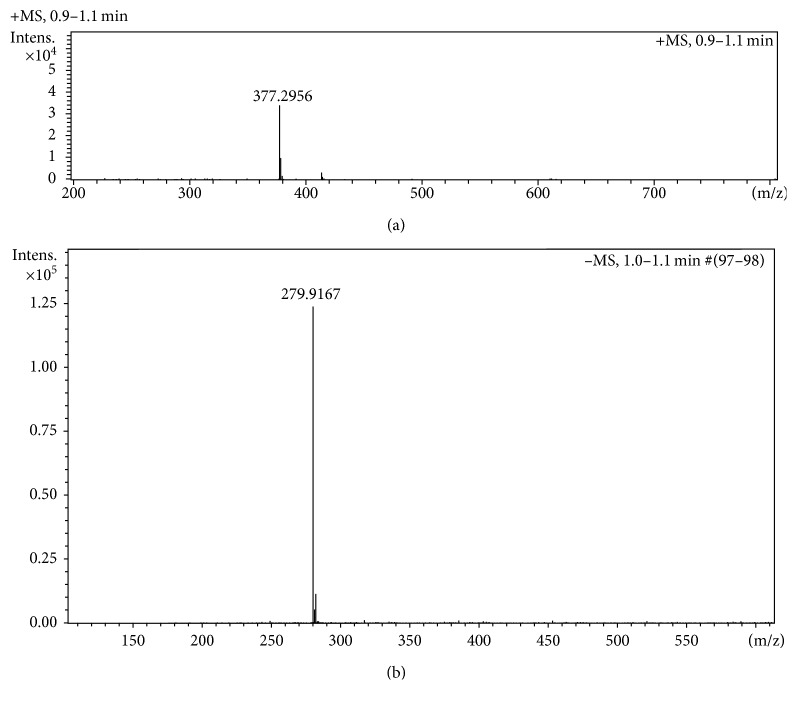
(a) MALDI MS+ and (b) MALDI MS for [C_26_H_37_N_2_][(CF_3_SO_2_)_2_N].

The matrix-assisted laser desorption/ionization (MALDI MS) spectra of [C_29_H_37_N_2_][(CF_3_SO_2_)N] were recorded for both the cation and anion targeting the cation and anion, respectively (Figure 2). The obtained molecular ion peaks for the cation appeared at *m*/*z* 377.2956 confirmed the quaternization of 1,10-phenanthroline with 1-bromotetradecane and the acquisition of the intended salt with the proposed formula (Figure 2(a)). Moreover, the recorded molecular ion peak for the anion appeared at m/e 279.9165 confirmed the successful anion exchange and isolation of pure salt (Figure 2(b)).

### 3.5. UV-Vis Spectra

**Figure 3:**
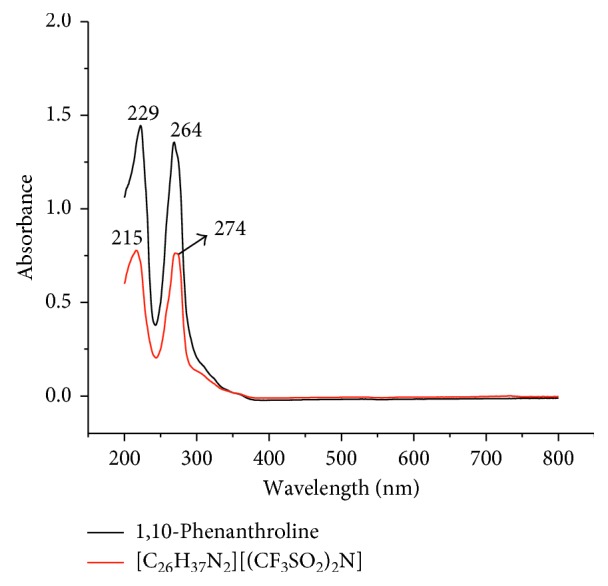
UV-Vis spectra of 1,10-phenanthroline and [C_26_H_37_N_2_][(CF_3_SO_2_)_2_N].

In addition to XPS, UV-Vis spectroscopy was employed to investigate the electronic environments of the final product and the starting of 1,10-phenanthroline to confirm the successful monoquaternization. The UV-Vis spectrum of 1,10-phenanthroline shows bands at 229 nm and 264 nm corresponding to *n* → *π* ^*∗*^ and *π* → *π* ^*∗*^ transitions, respectively. Following the quaternization, these bands appeared shifted to 215 nm and 274 nm, respectively (Figure 3). This is because, the quaternization involves the nonbonding electron in the bonding, thereby lowers the energy. The latter increases the energy gap between the nonbonding and the *π* ^*∗*^ orbitals. On the other hand, the quaternization develops a delocalized positive charge in the ring system that increases the energy of the *π* orbitals. Subsequently, it decreases the energy gap between *π* and *π* ^*∗*^ orbitals.

### 3.6. Thermal Properties

The response obtained from heating of the samples clearly demonstrated the influence of the anion on the nature of the salt produced. The cation is large in size over which the monopositive charge is highly delocalized over the entire aromatic portion. This makes it classified as a soft acid. The increase in the size of the counter mono-charged anion increases the extent of its softness [32]. In [C_26_H_37_N_2_]Br, the soft cation is coupled with a relatively hard anion which creates incompatibility in their interaction that made the salt relatively unstable. Because of this, it showed decomposition without melting, starting around 129.72°C. This is the consequence of the localized negative charge on the bromide that easily attacks the cation which creates relative instability [33]. On the other hand, the coupling of the soft cation with a relatively softer base in [C_26_H_37_N_2_][(CF_3_SO_2_)_2_N] resulted in thermally stable salt. This is expressed by its higher decomposition point which started at around 244°C (Table 2 and Supporting information 2(b) and 3). Furthermore, the cation and anion are both unsymmetrical that they hinder the crystalline packing of the salt. This fact significantly reduced the melting point to 48.77°C. The latter property makes it to be classified as a new ionic liquid, and the wide gap between its melting and decomposition temperatures gives [C_26_H_37_N_2_][(CF_3_SO_2_)_2_N], an attractive feature for potential applications such as electrochemistry [31, 34, 35] (Supporting information 2(b) and 3 and Table 2). Moreover, [C_26_H_37_N_2_][(CF_3_SO_2_)_2_N] typically demonstrated substantial supercooling as its freezing point is significantly lower than the melting point (−11.28 to 48.77°C) (Supporting information 3 and Table 2).

### 3.7. Antibacterial Screening

**Figure 4:**
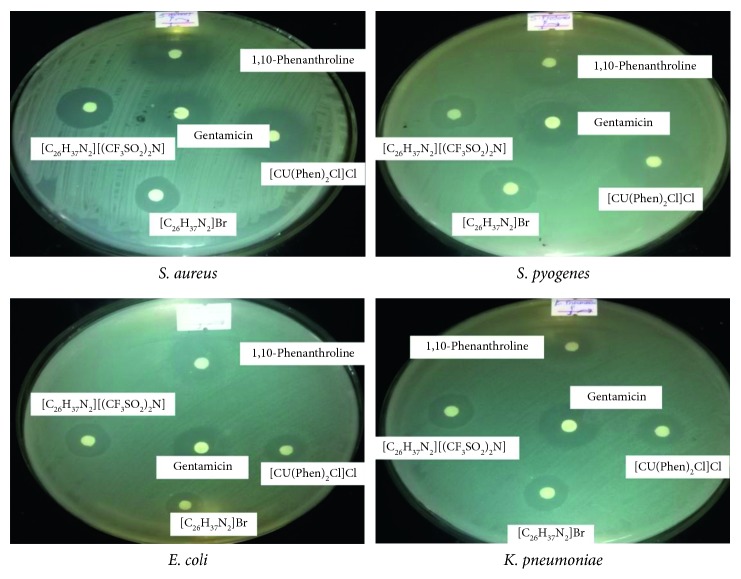
The inhibition observed by the actions of the salts.

The compounds were tested for their *in vitro* antimicrobial activity and were compared with the commercially available gentamicin. They were tested against two Gram-positive (*S. aureus* and *S. pyogenes*) and two Gram-negative (*E. coli* and *K. pneumoniae*) bacteria, and they all were found active against all the tested pathogens (Figure 4). The synthesized compounds, the metal complex, and gentamicin showed less activities than the molecular 1,10-phenanthroline against all the bacteria. The metal complex, [Cu(Phen)_2_Cl]Cl, showed better activities than the organic salts and gentamicin against the Gram-positive bacteria (*S. aureus* and *S. pyogenes*). On the other hand, the synthesized organic salts demonstrated better activities than [Cu(Phen)_2_Cl]Cl against the Gram-negative (*E. coli* and *K. pneumoniae*) bacteria (Figure 4 and Table 3). Furthermore, [C_26_H_37_N_2_][(CF_3_SO_2_)_2_N] is found better than its precursor, [C_26_H_37_N_2_]Br. The former statement is encouraging because these compounds succeeded in reaching the target passing two barriers of the Gram-negative bacteria, namely, the cell membrane and the cell wall of even the highly drug resistant *K. pneumoniae*. This is probably due to the very long and rod-like cation which contained relatively long lipophilic alkyl chain; its positive charge is highly delocalized throughout the aromatic ring portion; subsequently, it becomes amphiphilic which increased the cell permeability. Subsequently, the cell wall and cell membrane that surrounds the cell favors the passage of salts to reach to their target [36]. Moreover, significant biological activity differences are observed between [C_26_H_37_N_2_][(CF_3_SO_2_)_2_N] and [C_26_H_37_N_2_]Br. This is attributed to the better penetration of the former into the cellular target due to the lipophilicity of [(CF_3_SO_2_)_2_N]^−^. This is in very good agreement with the solubility experiment result that [C_26_H_37_N_2_][(CF_3_SO_2_)_2_N] is soluble only in organic solvents. The minimum inhibitory concentration (MIC) values of the salts are summarized in Table 4. The result shows that even though the organic salts show less inhibition zones than the metal complex against Gram-positive bacteria, they are found inhibiting at such very small minimum concentrations (Table 4).

## 4. Conclusions

The purity and synthesis of the intended salt was confirmed from the data obtained using all characterization techniques employed here. The results demonstrated that the effect of the anion on the properties of the salt is significant. This was revealed on their thermal and antibacterial results. The result of the *in vitro* biological activity studies indicated that the organic salts are biologically active against all the tested pathogens. This result makes them classified as a wide-range antibacterial agent. These organic salts are found better than the copper complex in their activities against the Gram-negative bacteria. This can be considered promising news as alternative drugs providing the cytotoxicity issue is resolved. This can lighten the concern of introducing metal ions into organisms. The DSC result showed that [C_26_H_37_ N_2_][(CF_3_SO_2_)_2_N] is an ionic liquid with large liquidus range.

## Figures and Tables

**Table 1:** CHNBrS elemental analysis measurements.

Compound	Elemental estimation				
	Calculated (found) (%)				
	H	C	N	Br	S
[C_26_H_37_N_2_]Br	8.10 (7.98)	68.27 (68.04)	6.13 (6.00)	17.51 (17.28)	—
[C_26_H_37_ N_2_][(CF_3_SO_2_)_2_N]	5.67 (5.64)	51.14 (51.01)	6.39 (6.28)	—	9.74 (9.59)

**Table 2:** Starting point and onset temperatures with the weight loss curves and melting and crystallization temperatures of the salts.

Compound	Temperature (°C)				
	Start	Onset	Glass transition	Crystallization	Melting
[C_26_H_37_N_2_]Br	129.72	157.41	—	—	—
[C_26_H_37_N_2_][(CF_3_SO_2_)_2_N]	244.41	290.31	−0.60	−11.28	48.77

**Table 3:** Antibacterial studies of the investigated compounds (inhibition zones).

Compound	Inhibition zone (mm)			
	Gram-negative bacteria		Gram-positive bacteria	
	*E. coli*	*K. pneumoniae*	*S. aureus*	*S. pyogenes*
[C_26_H_37_ N_2_]Br	19.75	19.75	16.50	25.00
[C_26_H_37_ N_2_][(CF_3_SO_2_)_2_N]	20.25	20.25	23.50	20.25
Gentamicin	31.25	25.00	26.50	28.25
[Cu(Phen)_2_Cl]Cl	15.00	17.25	29.75	29.50
1,10-Phenanthroline	36.00	18.75	36.00	31.25

**Table 4:** MIC assays of the salts against four bacterial pathogens.

Compound	Minimum concentration of microorganism growth (*μ*g/mL)			
	Gram-negative bacteria		Gram-positive bacteria	
	*E. coli*	*K. pneumoniae*	*S. aureus*	*S. pyogenes*
[C_26_H_37_ N_2_]Br	50	50	50	50
[C_26_H_37_ N_2_][(CF_3_SO_2_)_2_N]	50	75	75	75
[Cu(Phen)_2_Cl]Cl	100	125	75	75

## Data Availability

The authors are not making their data available at this time.

## Supplementary Materials

Supplementary Materials The supporting information contained NMR (^1^H, ^13^C, and ^31^F) (Supporting information 1(a)–1(e)), thermograms and thermogravimetric analysis (TGA) curves for [C_26_H_37_ N_2_] and [C_26_H_37_ N_2_][(CF_3_SO_2_)_2_N] (Supporting information 2(a) and 2(b)), and differential scanning calorimetric curve for [C_26_H_37_ N_2_][(CF_3_SO_2_)_2_N] (Supporting information 3).
