# Supplementary material for: Synthesis of N-Tetradecyl-1,10-phenathrolinium-Based New Salts for Biological Applications
Source: Bioinorg Chem Appl. 2018 Jul 17;2018:8097483. doi: 10.1155/2018/8097483 (PMC6079542; doi:10.1155/2018/8097483)
Supplement: Supplementary Materials — The supporting information contained NMR (1H, 13C, and 31F) (Supporting information 1(a)–1(e)), thermograms and thermogravimetric analysis (TGA) curves for [C26H37 N2] and [C26H37 N2][(CF3SO2)2N] (Supporting information 2(a) and 2(b)), and differential scanning calorimetric curve for [C26H37 N2][(CF3SO2)2N] (Supporting information 3). [file 8097483.f1.pdf]

Supporting Information

NMR Spectra

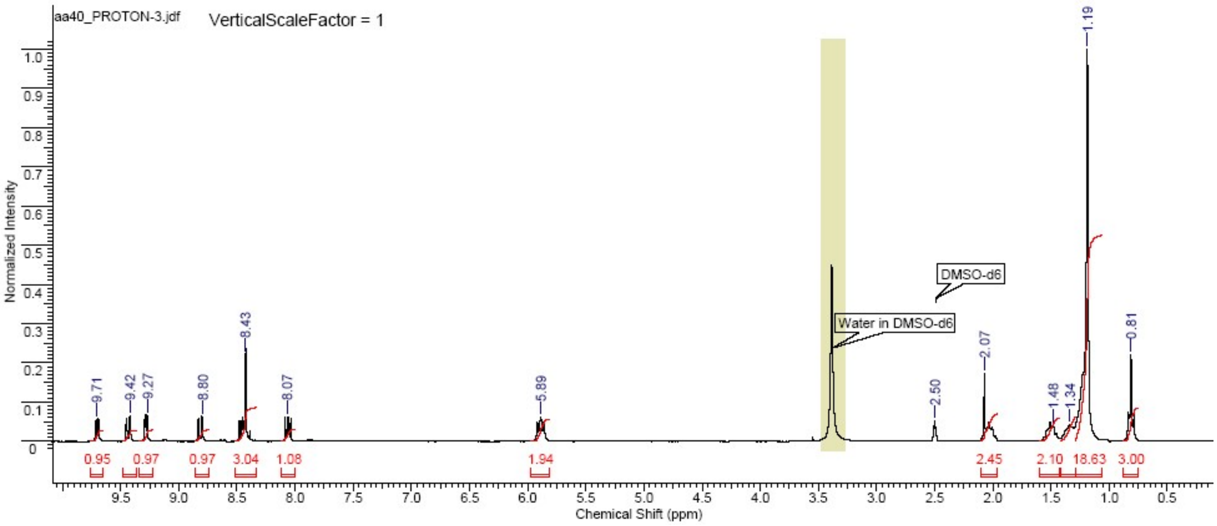

a

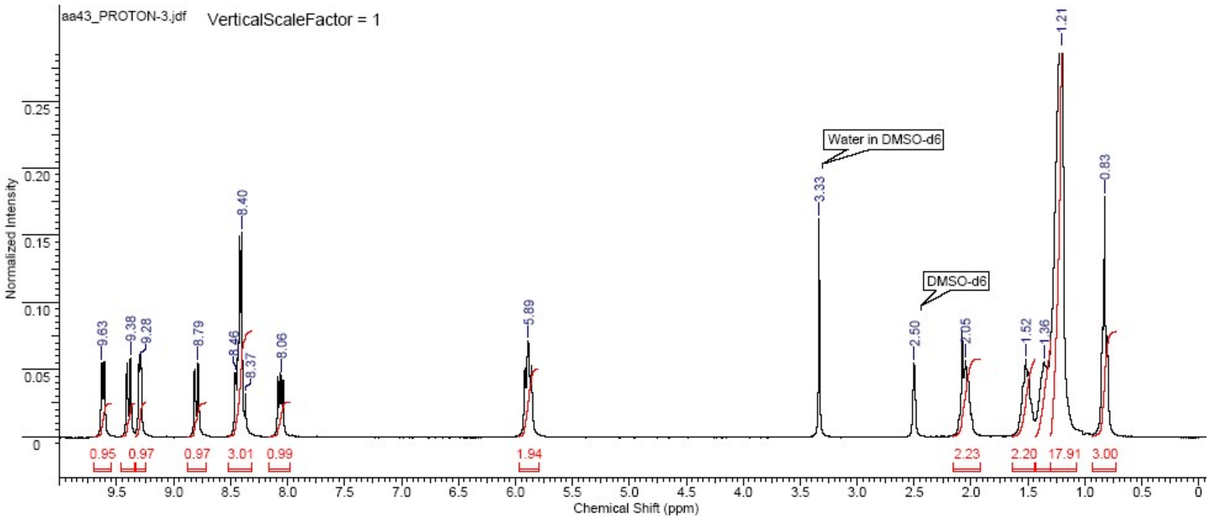

b

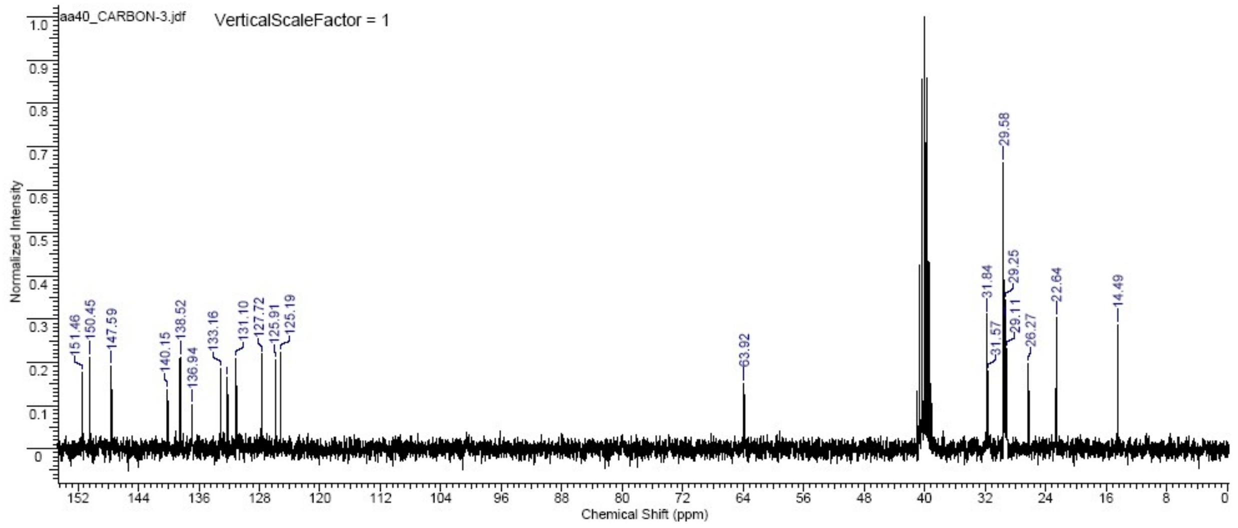
aa40_CARBON-3.jdf
VerticalScaleFactor = 1
151.46
150.45
147.59
140.15
138.52
136.94
133.16
131.10
127.72
125.91
125.19
63.92
31.84
31.57
29.58
29.25
29.11
26.27
22.64
14.49
Normalized Intensity
Chemical Shift (ppm)

c

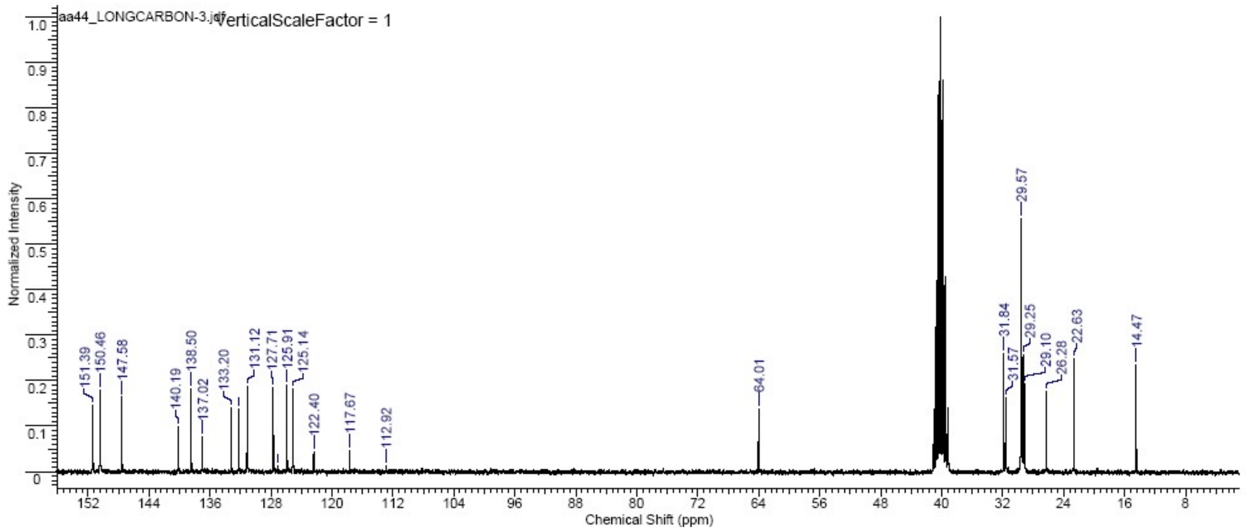
aa44_LONGCARBON-3.jdf
VerticalScaleFactor = 1
151.39
150.46
147.58
140.19
138.50
137.02
133.20
131.12
127.71
125.91
125.14
122.40
117.67
112.92
64.01
31.84
31.57
29.57
29.25
29.10
26.28
22.63
14.47
Normalized Intensity
Chemical Shift (ppm)

d

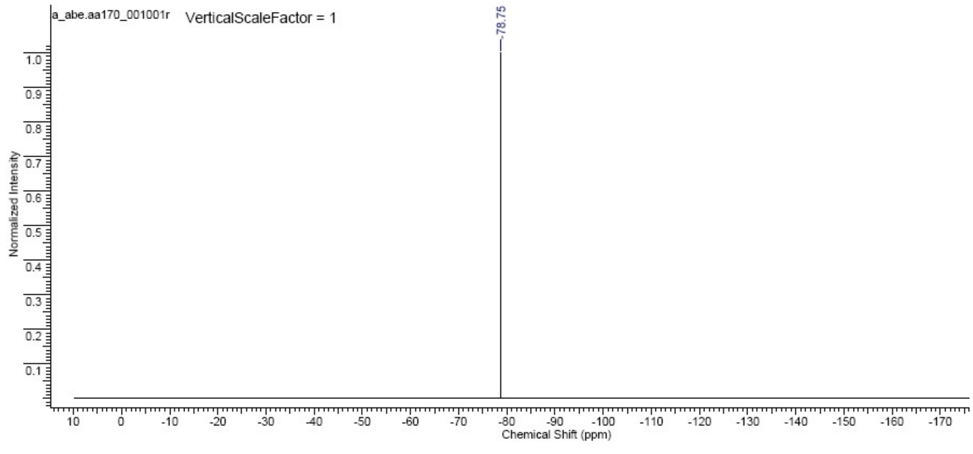
a_abe.aa170_001001r
VerticalScaleFactor = 1
-78.75
Normalized Intensity
Chemical Shift (ppm)

e

Figure 1: a) ^{1}H NMR of $[C_{26}H_{37}N_2]Br$, b) ^{1}H NMR of $[C_{26}H_{37}N_2][(CF_3SO_2)_2N]$, c) ^{13}C NMR of $[C_{26}H_{37}N_2]Br$, d) ^{13}C NMR of $[C_{26}H_{37}N_2][(CF_3SO_2)_2N]$, e) ^{19}F NMR of $[C_{26}H_{37}N_2][(CF_3SO_2)_2N$

Thermograms

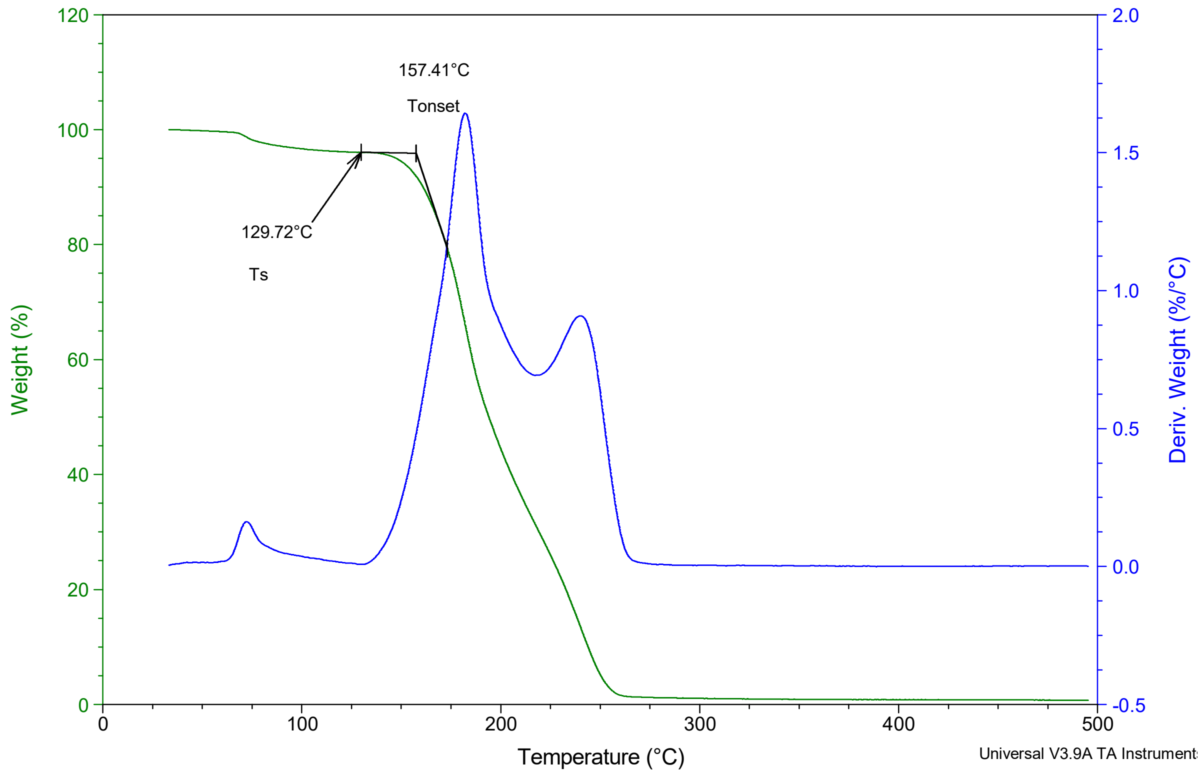

a

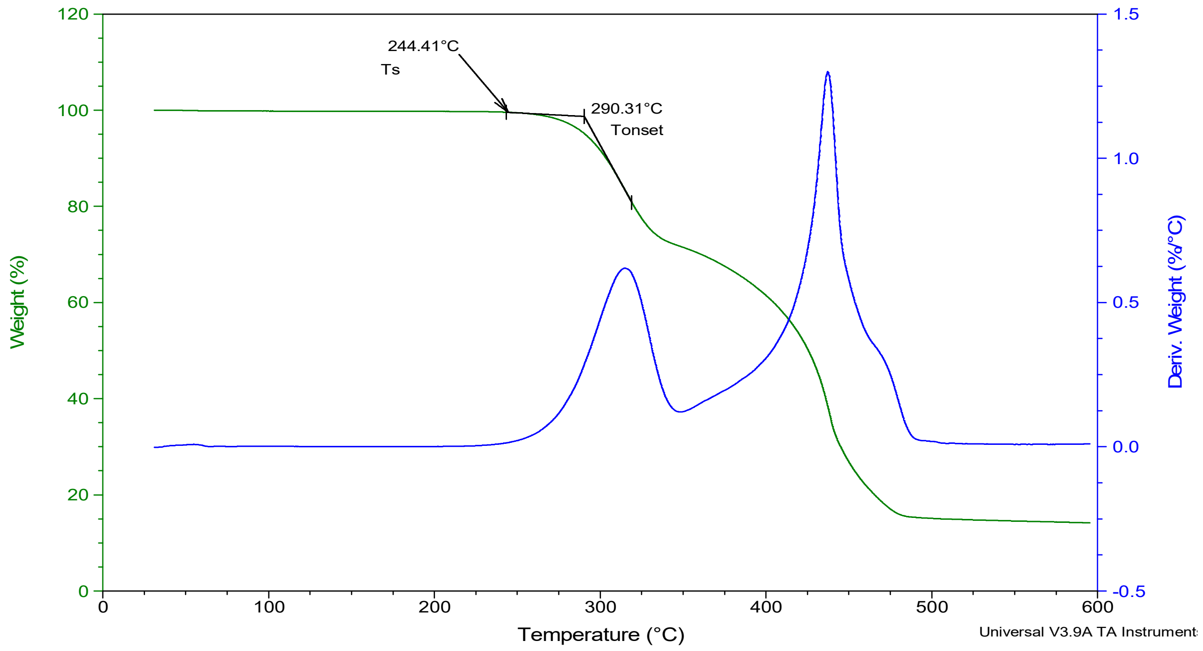

B

Figure 2: Characteristic decomposition curve determined by TGA, indicating the start and onset temperatures of: a) [$C_{26}H_{37}N_2$]Br, b)[$C_{26}H_{37}N_2$][$(CF_3SO_2)_2N$]

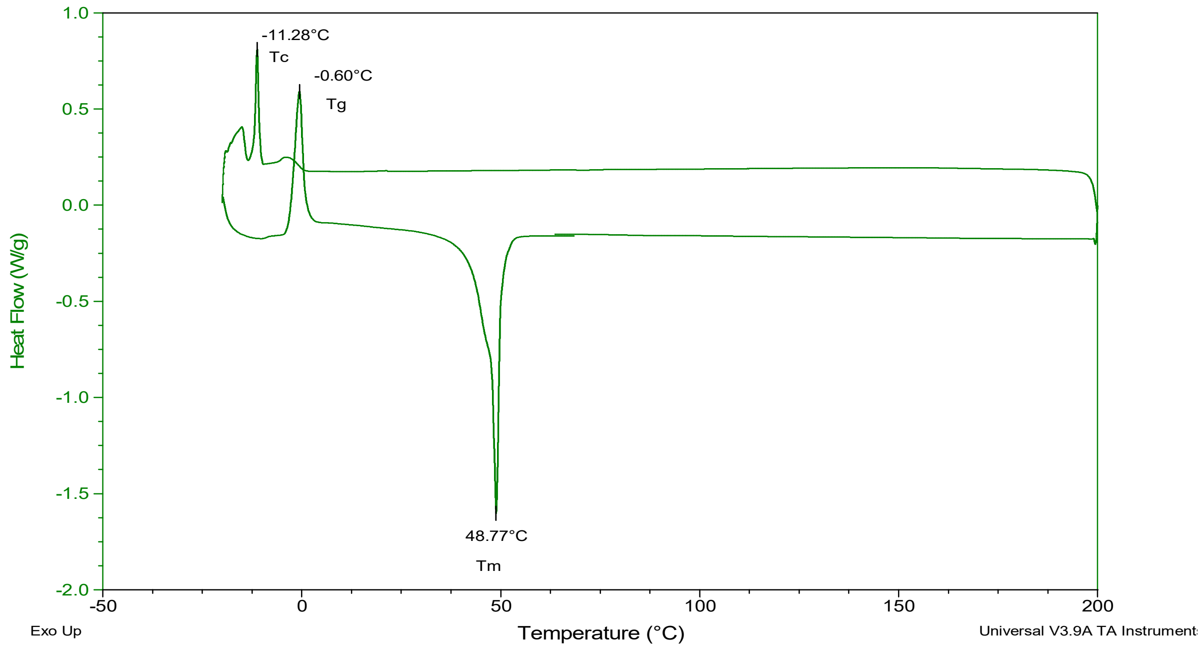

Figure 3: Characteristic phase transition of [$C_{26}H_{37}N_2$][$(CF_3SO_2)_2N$], determined by DSC, indicating the melting and crystallization temperatures of [$C_{26}H_{37}N_2$][$(CF_3SO_2)_2N$]
